# Supplementary material for: Cholesterol Homeostasis Modulates Platinum Sensitivity in Human Ovarian Cancer
Source: Cells. 2020 Mar 30;9(4):828. doi: 10.3390/cells9040828 (PMC7226826; doi:10.3390/cells9040828)
Supplement: Supplementary file 1 [file cells-09-00828-s001.zip › Supplementary Figures.docx]

Supplementary Figures


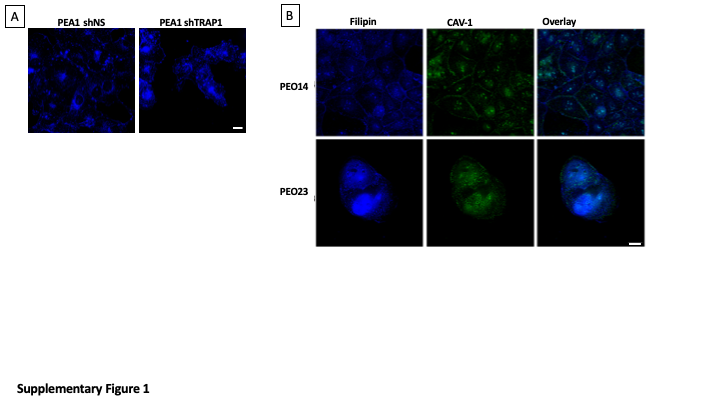


**Figure S1.** Analysis of membrane cholesterol in chemoresistant cells. (**a**) Microscopy analysis of unesterified cholesterol through the visualization of filipin fluorescence stable TRAP1 knockdown clones in cisplatin-sensitive PEA1 cells (shTRAP1) compared to the non-silencing shRNA clones (shNS). Maximum projection of Z-slices is shown. Scale bar = 20 μm. (**b**) Representative images of double staining, in cisplatin-sensitive PEO14 and in the matched cisplatin-resistant PEO23 cells, of unesterified cholesterol (Filipin) with Caveolin-1 (CAV-1). Scale bar = 20 μm.


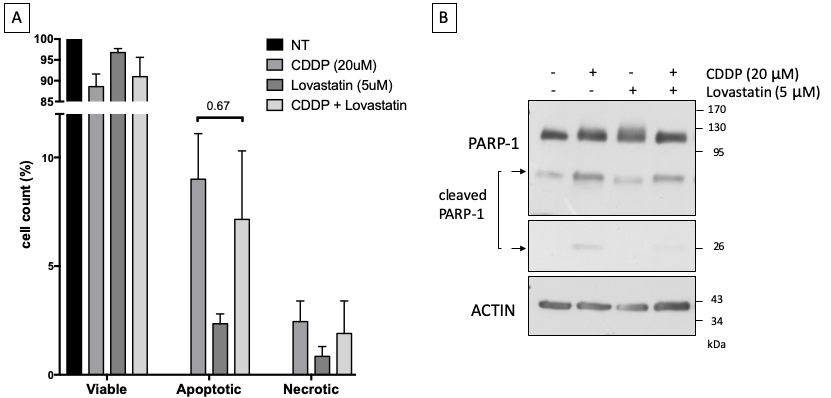


**Figure S2.** Lovastatin protects from cisplatin-induced apoptosis. (**a**) Cytofluorimetric analysis of cisplatin-sensitive PEA1 ovarian cancer cells marked with Annexin V and Propidium Iodide. 24 h after seeding, cells were treated with lovastatin (5 μM) for 24 h and then treated with cisplatin (CDDP, 20 μM) for additional 48 h. Data are expressed as mean ± S.E.M. from two independent experiments. Number above bars represents the statistical significance (*p*-value) based on the two-tailed Student’s t-test. (**b**) PEA1 cells treated as described in (**a**) were collected, equal amounts of total lysates were loaded for SDS-PAGE, transferred on a PVDF membrane and immunoblotted with indicated antibodies.
